# Gall Bladder And Common Bile Duct Stones – When Is Direct Cholangiography Indicated?

**DOI:** 10.1155/1989/51967

**Published:** 1989

**Authors:** B. M. L. Kapur, M. C. Mishra, P. S. V. Rao, R. K. Tandon

**Affiliations:** All India Institute of Medical Sciences Ansari Nagar New Delhi 110029 India

## Abstract

The medical records of 277 consecutive patients who underwent cholecystectomy for benign gall stone disease, were reviewed to determine the incidence and cause of biliary tract obstructuion.

Obstructive jaundice (icteric obstructive biliopathy) was present in 38 cases. This was due to choledocholithiasis in 22, Mirizzi's Syndrome in two, biliobiliary fistula in eight and biliary stricture in five patients. Preoperative direct cholangiography (ERCP/PIC) was helpful.

Anicteric patients were classified on the basis of a history of jaundice, serum alkaline phosphatase, sonography and operative findings. Anicteric patients with evidence of biliary tract pathology (anicteric obstructive biliopathy) had a significant incidence of choledocholithiasis (33.3% ). Biliary complications were uncommon in this group (4.3%). Peroperative cholangiography was carried out and was valuable in these patients but was normal in all 83 patients who had no evidence of biliary obstruction.


*HPB Surgery* 1989, Vol. 1, pp. 201–205
Reprints available directly from the publisher
Photocopying permitted by license only

© 1989 Harwood Academic Publishers GmbH
Printed in Great Britain

### GALL BLADDER AND COMMON BILE DUCT STONES - WHEN IS DIRECT CHOLANGIOGRAPHY INDICATED?

B.M.L. KAPUR*, M.C. MISHRA, P.S.V. RAO, and R.K. TANDON

*All India Institute of Medical Sciences, New Delhi-110029, India*

The medical records of 277 consecutive patients who underwent cholecystectomy for benign gall stone disease, were reviewed to determine the incidence and cause of biliary tract obstructuion.

Obstructive jaundice (icteric obstructive biliopathy) was present in 38 cases. This was due to choledocholithiasis in 22, Mirizzi's Syndrome in two, biliobiliary fistula in eight and biliary stricture in five patients. Preoperative direct cholangiography (ERCP/PIC) was helpful.

Anicteric patients were classified on the basis of a history of jaundice, serum alkaline phosphatase, sonography and operative findings. Anicteric patients with evidence of biliary tract pathology (anicteric obstructive biliopathy) had a significant incidence of choledocholithiasis (33.3%). Biliary complications were uncommon in this group (4.3%). Peroperative cholangiography was carried out and was valuable in these patients but was normal in all 83 patients who had no evidence of biliary obstruction.

KEY WORDS: Obstructive biliopathy, Extrahepatic bileduct obstruction, Cholelithiasis, Choledocholithiasis, Cholecystectomy, Cholangiography.

### INTRODUCTION

This paper is concerned with a spectrum of benign obstructive disorders of the biliary tract associated with gallstone disease. In addition to biliary obstruction due to choledocholithiasis and associated inflammation it includes complications of gallstones such as Mirizzi's syndrome, biliobiliary fistula and biliary stricture. In Mirizzi's syndrome, a stone impacted in the cystic duct or neck of the gall bladder compresses and obstructs the common (hepatic/bile) duct. If untreated, the stone may eventually erode into the common duct creating a biliobiliary fistula. The stone(s) usually lies astride the fistula and obstructs the common duct. Trauma and infection secondary to stones in the common duct can lead to stricture formation and obstruction.

In this study we present the relative incidence of various causes of benign obstructive biliopathy in a consecutive series of patients operated upon for gall stones.

* Address correspondence to: Dr. B.M.L. Kapur, Professor of Surgery, All India Institute of Medical Sciences, Ansari Nagar, New Delhi-110029, India

### PATIENTS AND METHODS

The records of 277 consecutive patients who underwent cholecystectomy for gallstone disease in a single surgical unit at this Institute over the past 3 years were reviewed. Those with a malignant lesion of the biliary tract were excluded, even if there was associated cholelithiasis. Cases of haemolytic anaemia with cholelithiasis were also excluded.

All patients had serum bilirubin and alkaline phosphatase estimations as well as peroperative cholangiography. A preoperative sonographic examination of the biliary system was done in 171 patients. The rest had undergone cholecystectomy during the earlier part of the study, before sonography became a routine investigation.

The patients were divided into three groups:

Group I: Patients with jaundice or raised serum bilibrium at admission.

Group II: Anicteric patients with a history of jaundice and biliary pain.

Group III: Anicteric patients having no history of jaundice with biliary pain.

The operative findings in these groups were analysed for choledocholithiasis and associated complications (Table) and correlated with biochemical and sonographic findings.

### RESULTS

*Group I:* In 38 patients with jaundice, all but one had stones in the common (hepatic/bile) duct at surgery. Choledocholithiasis was the only cause of jaundice in 22 patients. The other 15 (40%) patients had choledocholithiasis, complicated by Mirizzi's syndrome in two (one had an intrahepatic gall bladder), biliobiliary fistula in eight, and high/low biliary strictures in one and four patients respectively.

*Group II:* Thirty three anicteric patients had a history of jaundice with biliary pain. Twenty three had no operative or cholangiographic evidence of choledocholithiasis. The other ten (30.2%) had choledocholithiasis, and this was associated with a low biliary stricture in two patients and a biliobiliary fistula in one patient.

*Group III:* 206 anicteric patients had no history of jaundice. Of them 190 had no stones or obstruction in the common duct. Uncomplicated choledocholithiasis was present in 15 patients. One patient had choledocholithiasis with a low biliary stricture.

*Serum Alkaline Phosphatase:* The serum alkaline phosphatase was raised in all 38 patients with jaundice (group I). It was also raised in 34 anicteric patients (seven from group II and 27 from group III). Choledocholithisis was present in seven of these patients (three from group II and four from group III) including a patient with a biliobiliary fistula.

*Sonography:* Sonography examination of 113 patients of group III showed evidence of common duct stones or obstruction in six. At surgery, four had common duct stones without any clinical evidence or complications. Sonographic examination of 20 patients of group II showed evidence of choledocholithiasis in two patients and this was confirmed at surgery.

*Obstructive biliopathy in anicteric patients:* 29 patients in group III had raised serum alkaline phosphatase and/or sonographic evidence of common duct stones or obstruction. Seven other patients of this group had an operative indication for

**Table 1**

| | | *No. of Patients* | *Gall stone disease without choledoch-olithiasis* | *Gall stone disease with choledocho-lithiasis* | *Gall stone disease with complications* | | | | |
|---|---|---|---|---|---|---|---|---|---|
| | | | | | *Mirizzi's* | *Bilio Biliary Fistula* | *High Biliary stricture* | *Low Biliary stricture* | *Total* |
| Group I | (Icteric at admission) | 38 (13.7%) | 1 | 22 (58%) | 2 | 8* | 1 | 4 | 15 (40%) |
| Group II | (Anicteric with history of Jaundice) | 33 (12%) | 23 | 7 | – | 1 | – | 2 | 3 (9%) |
| Group III | Anicteric with no history of Jaundice | 206 (74.3%) | 190 | 15 (7.28%) | – | – | – | 1 | 1 (0.49%) |
| TOTAL | | 277 | 214 | 44 | 2 (0.7%) | 9 (3.25%) | 1 (0.36%) | 7 (2.5%) | 19 (6.9%) |

* 2 had high biliary strictures also peroperative cholangiography, (choledochal dilatation in 5, multiple small gall stones with wide cystic duct in 2). All these patients had choledocholithiasis on exploration. Thus historical, biochemical, sonographic or operative evidence of biliary obstruction was present in 69 anicteric patients (all the 33 patients of group II + 36 patients of group III). Of these 23 patients had choledocholithiasis including the three patients with biliary complications. There was no historical, biochemical, sonographic or operative evidence of biliary obstruction in 83 anicteric patients. The peroperative cholangiogram did not demonstrate stones in any. 87 anicteric patients, including two with uncomplicated choledocholithiasis and one with low biliary stricture could not be classified due to the absence of sonographic data.

### DISCUSSION

There is a high (40%) incidence of biliary complications in our patients with choledocholithiasis and obstructive jaundice. This may be related to late presentation of the patients for surgical treatment. Preoperative detection of these complications is important. A biliobiliary fistula usually contains a large impacted stone, and is surrounded by dense sclerosis. This hard lump may be mistaken for a tumor at surgery. Further in the presence of a biliobiliary fistula, the technique for cholecystectomy is modified to avoid injury to the upper biliary tract [1]. Both antegrade and retrograde dissection should be avoided. Instead an incision should be made over the inferior surface of the gall bladder, the stones removed and the gall bladder debrided and repaired over a T tube or anastamosed to the duodenum. preoperative detection of biliary stricture helps in planning the appropriate operation. In the presence of biliary complications of gall stones, the biliary anatomy is often distorted. Hence proper preoperative delineation of the biliary tract helps identify structures at surgery and detection of anomalies. Preoperative detection of biliary complications of gallstones disease is possible by direct (endoscopic or percutaneous) cholangiography. Preoperative direct cholangiography done in the radiology department is superior to peroperative cholangiography in detecting these complications and in delineating the biliary tract. It also helps to plan the operation in advance and saves operating time. Hence, it is desirable to get preoperative direct cholangiography in these patients even if the cause of jaundice has been established as gallstones by other investigations. But in accordance with others [2] in the great majority of cases (of choledocholithiasis with obstructive jaundice) the clinical features of pain and recurrent jaundice, together with the findings on oral cholecystography and ultrasound, give the surgeon enough information to warrant laparotomy with a firm preoperative diagnosis of calculous biliary disease.

In the 69 patients with only historical, biochemical, sonographic or operative evidence of biliary obstruction, there was a significant incidence (33.3%) of stones in the common bile duct but the incidence of biliary complications of gallstones was low (4.3%). Preoperative cholangiogram in these patients is not necessary and peroperative cholangiography should suffice.

In 83 patients who had no pre-or intra-operative evidence of biliary obstruction or choledocholithiasis, there was no incidence of either biliary complications or common bile duct stones on routine peroperative cholangiography. Hence, it can be argued that there is no need to obtain pre or peroperative cholangiogram in this group. Indeed Del Santo et al [3] have recently shown that if there was no history of jaundice or any clinical indication for common bile duct exploration and serum bilirubin, alkaline phosphatase, SGOT and lactate dehydrogenase were normal, there was no incidence of choledocholithiasis on peroperative cholangiography. Similarly in 249 patients who had no clinical indications for exploration, Mofti et al[4] found that none had positive cholangiographic findings. These arguments for the selective use of peroperative cholangiogram have however, been challenged by others [5]. Blumgart [6] has advocated the use of routine peroperative cholangiography in all cases of gallstones disease so that any anomaly in the biliary tract may be detected. This may help avoid injury to the extrahepatic biliary tree.

Thus, gall stone disease can be classified as:

1) Icteric obstructive gallstone biliopathy: when associated with obstructive jaundice.
2) Anicteric Obstructive gallstone biliopathy: when there is any other evidence of biliary obstruction:
   a) Historical: Past history of jaundice with biliary colic;
   b) Biochemical: raised serum alkaline phosphatase;
   c) Sonographic: evidence of common bile duct stones or obstruction;
   d) Operative: palpable choledochal stones, dilated biliary tract.
3) Non obstructive gall stone disease: When there is no historical, biochemical, sonographic or operative evidence of biliary obstruction.

Patients with icteric obstructive biliopathy (38 patients) had a high incidence (40%) of biliary complications of gallstones. Preoperative direct cholangiography (ERCP/PIC) was helpful in them. Patients with anicteric obstructive biliopathy had a significant incidence of choledocholithiasis (33.3%) but biliary complications were uncommon (4.3%) and a peroperative cholangiogram was sufficient in them. On the other hand the peroperative cholangiogram was normal in all the 83 patients who had no historical, biochemical, sonographic or operative evidence of biliary obstruction.

### *References*

1. Corlette, M.B. and Bismuth, H. Biliobiliary fistula: A trap in the surgery of cholelithiasis. Arch Surg. 1975, 110: 377–383.
2. Ellis, H. Choledocholithiasis. In: Maingot's Abdominal Operations. Edited by S.I. Schwartz and H. Ellis. pp. 1883–1907, Norwalk, Connecticut: Appleton Century Crofts, 1985.
3. Del Santo, P., Kazarian, K.K., Rogers, J.F., Bevins, P.A. and Hall, J.R.: Prediction of operative cholangiography in patients undergoing elective cholecystectomy with routine liver function chemistries. Surg. 1985, 98: 7–11.
4. Mofti, A.B., Ahmed, I., Tandon, R.C., Al-Tameem, M.M. and Al-Khudiary, N.N.: Routine or Selective peroperative cholangiography. Br. J. Surg. 1986, 73: 548-550.
5. McCarthy, J.D., Surgical Pros and Cons (Editorial) Surg. Gynecol Obstet 1981, 153: 250.
6. Kelley, C.J., Blumgart, L.H. Peroperative Cholangiography and postcholecystectomy biliary strictures. Ann R. Coll Surg. Engl. 1985, 67: 93–95.

*Accepted by S. Bengmark.*

